# Direct Nasal Swab for Rapid Test and Saliva as an Alternative Biological Sample for RT-PCR in COVID-19 Diagnosis

**DOI:** 10.1128/spectrum.01998-22

**Published:** 2022-12-01

**Authors:** Saiful Arefeen Sazed, Mohammad Golam Kibria, Md Fahad Zamil, Mohammad Sharif Hossain, Jeba Zaman Khan, Rifat Tasnim Juthi, Mohammad Enayet Hossain, Dilruba Ahmed, Zannatun Noor, Rashidul Haque, Mohammad Shafiul Alam

**Affiliations:** a International Centre for Diarrhoeal Disease Research Bangladesh (icddr,b), Mohakhali, Bangladesh; University of Sussex

**Keywords:** COVID-19, rapid diagnostic tests, SARS-CoV-2, salivary specimen, saliva, RT-PCR

## Abstract

Accurate and early diagnoses are prerequisites for prompt treatment. For coronavirus disease 2019 (COVID-19), it is even more crucial. Currently, choice of methods include rapid diagnostic tests and reverse transcription polymerase chain reaction (RT-PCR) using samples mostly of respiratory origin and sometimes saliva. We evaluated two rapid diagnostic tests with three specimen types using viral transport medium (VTM) containing naso-oropharyngeal (NOP) swabs, direct nasal and direct nasopharyngeal (NP) samples from 428 prospective patients. We also performed RT-PCR for 428 NOP VTM and 316 saliva samples to compare results. The sensitivity of the SD Biosensor Standard Q COVID-19 antigen (Ag) test kit drastically raised from an average of 65.55% (NOP VTM) to 85.25% (direct nasal samples), while RT-PCR was the gold standard. For the CareStart kit, the sensitivity was almost similar for direct NP swabs; the average was 84.57%. The specificities were ≥95% for both SD Biosensor Standard Q and CareStart COVID-19 Ag tests in all platforms. The kits were also able to detect patients with different variants as well. Alternatively, RT-PCR results from saliva and NOP VTM samples showed high sensitivities of 96.45% and 95.48% with respect to each other as standard. The overall results demonstrated high performance of the rapid tests, indicating the suitability for regular surveillance at clinical facilities when using direct nasal or direct NP samples rather than NOP VTM. Additionally, the analysis also signifies not showed that RT-PCR of saliva can be used as an choice of method to RT-PCR of NOP VTM, providing an easier, non-invasive sample collection method.

**IMPORTANCE** There are several methods for the diagnosis of coronavirus disease 2019 (COVID-19), and the choice of methods depends mostly on the resources and level of sensitivity required by the user and health care providers. Still, reverse transcription polymerase chain reaction (RT-PCR) has been chosen as the best method using direct naso-oropharyngeal swabs. There are also other methods of fast detection, such as rapid diagnostic tests (RDTs), which offer result within 15 to 20 min and have become quite popular for self-testing and in the clinical setting. The major drawback of the currently used RT-PCR method is compliance, as it may cause irritation, and patients often refuse to test in such a way. RDTs, although inexpensive, suffer from low sensitivity due to technical issues. In this article, we propose saliva as a noninvasive source for RT-PCR samples and evaluate various specimen types at different times after infection for the best possible output from COVID-19 rapid tests.

## INTRODUCTION

Coronavirus disease 2019 (COVID-19) has already tormented us with over 517 million cases and 6.2 million deaths (1). There have been vaccines out there to tackle the severity of the circulating variants. Medicines such as molnupiravir and remdesivir have been approved under emergency use authorization (EUA) only; yet, millions of people are being infected by severe acute respiratory syndrome coronavirus 2 (SARS-CoV-2) (2, 3). New variants such as Omicron, IHU, etc., have emerged, and it may take a significant amount of time before we are free of COVID-19 worldwide (4, 5).

The detection of COVID-19 has been very sensitive and has improved over the time (6). Reverse transcription polymerase chain reaction (RT-PCR) from naso-oropharyngeal (NOP) swabs is considered the gold standard for detection of COVID-19 (7). Recently, rapid diagnostic tests (RDTs), well known for being fast, have been questioned, as several studies suggest variable diagnostic sensitivity (8). RDTs are popular in community and clinical settings (9). Hence, RDTs need more attention in terms of evaluation of the existing kits and choice of route of sample collection (10). On the other hand, the major flaws of NP swab collection process itself is invasive and sometimes not feasible for noncooperative patients (11). Some patients coming to clinical facilities for COVID-19 testing often sneeze due to irritation inside the nostrils. During nasal or NP specimen swab collection, there is a possibility of droplet or aerosol generation through such a process that may infect the health care professional or other uninfected patients (12). A notable advantage of detection of COVID-19 through RT-PCR of saliva is that it reduces the chances of spreading severe acute respiratory syndrome coronavirus 2 (SARS-CoV-2), thereby keeping others safe.

**FIG 1:**
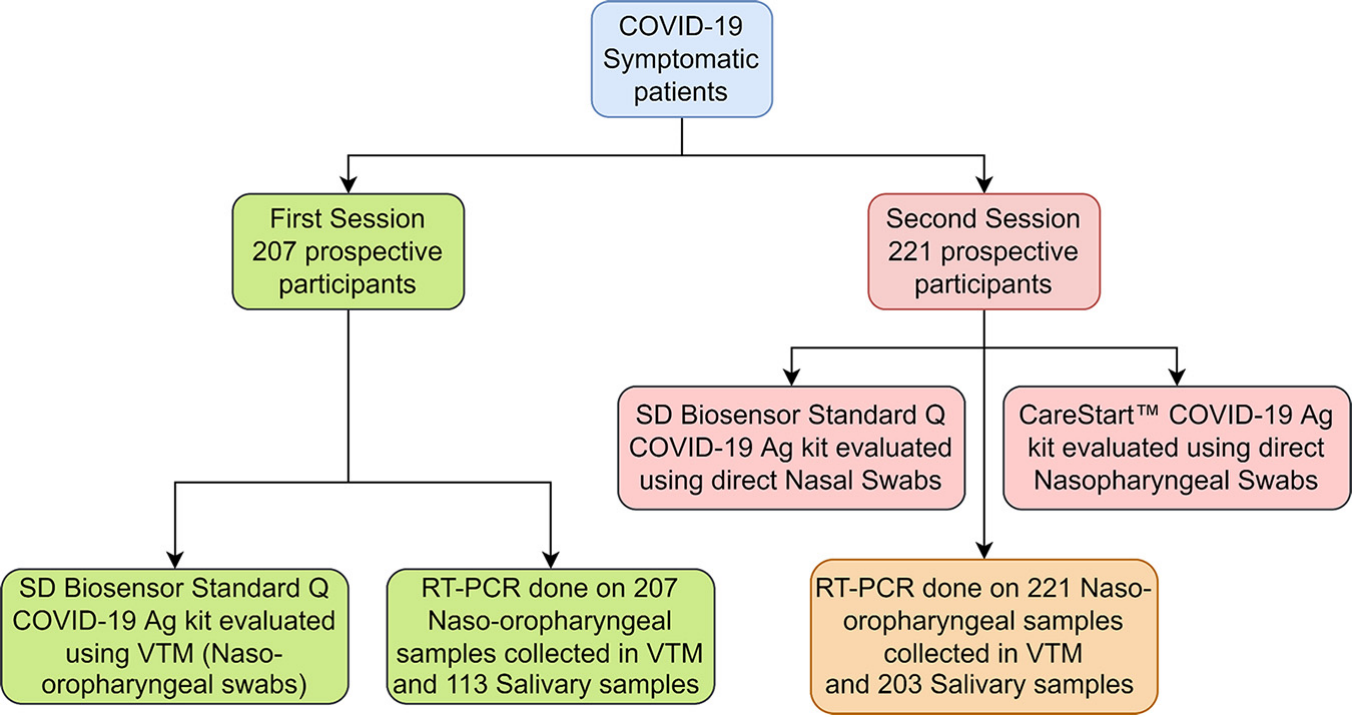
Flowchart showing the sample size and the steps of rapid testing and RT-PCR of VTM and saliva in two COVID-19 waves.

Overall, current diagnostic methods need to be assessed to offer insights into the most practical and convenient choice among the existing methods. In this study, we evaluated multiple rapid diagnostic kits with different sample types and reevaluated the gold standard method in terms of sample sources, such as those of naso-oropharyngeal and salivary origin (Fig. 1).

## RESULTS

### Enrollment.

**FIG 2:**
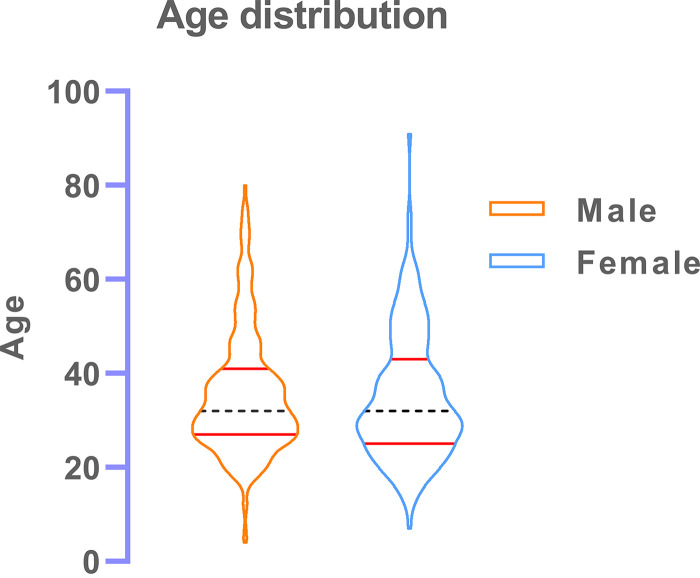
A violin curve depicting the distribution of age among the symptomatic male and female patients coming for COVID-19 RT-PCR testing.

A total of 428 patients were enrolled in two sessions with 269 males (62.85%; mean of 35.42, median age [interquartile range {IQR}] of 32 [27 to 41]) and 159 females (37.15%; mean of 34.97, median age [IQR] of 32 [25 to 43]). The age group description is depicted in Fig. 2.

### RT-PCR results for naso-oropharyngeal swabs in viral transport medium (VTM).

Of 428 patients, 268 (62.62%) tested positive by RT-PCR, and the cycling threshold (*C_T_*) values ranged from 10.44 to 34.86 for the nucleocapsid 1 (N1) gene (mean ± standard deviation [SD]: 19.96 ± 5.66) and 11.4 to 37.87 for N2 (mean ± SD: 20.79 ± 5.81), respectively. There was no mean significant difference between the N1 and N2 *C_T_* values (*P* > 0.05). The high and low positives were included in the study, as depicted in Table 1.

**TABLE 1:** Frequency distribution of 268 NOP VTM RT-PCR-positive samples with *C_T_* values for N1 and N2 targets

*C_T_* values	Frequency	
	N1	N2
<18	110	101
18 to <22	72	69
22 to <26	42	52
≥26	44	46
Total	268	268

### Performance evaluation of RT-PCR in terms of sample type.

316 salivary specimens were collected in both sessions out of a total of 428 patients. Only 113 samples were collected in the first session, as a very limited number of saliva samples could be obtained from patients who were willing to participate. The analysis was performed in terms of sample type. Nasal samples collected in VTM were considered the gold standard for evaluation of the salivary samples, and vice versa was also performed. Despite the sample source or collection method, RT-PCR was used for both cases. Salivary samples had a sensitivity of 96.45% (95% confidence interval [95% CI] of 92.82% to 98.56%) and specificity of 92.44% (95% CI of 86.13% to 96.48%) when VTM samples were considered the gold standard. The positive predictive value (PPV) and negative predictive value (NPV) were 95.48% (95% CI of 91.84% to 97.54%) and 94.02% (95% CI of 88.34% to 97.02%), respectively. In this case, 7 RT-PCR-positive samples of VTM sources were negative by RT-PCR using corresponding salivary sources.

On the other hand, RT-PCR results of VTM samples were analyzed considering the salivary sample RT-PCR results as the gold standard. The sensitivity was almost similar to salivary samples, that is, 95.48% (95% CI of 91.59% to 97.91%), and specificity was 94.02% (95% CI of 88.06% to 97.56%). The PPV and NPV were 96.45% (95% CI of 92.97% to 98.24%) and 92.44% (95% CI of 86.57% to 95.86%), respectively. Here, 9 RT-PCR-positive samples from salivary sources were found to be negative by RT-PCR using corresponding VTM sources. In both cases, similar high agreement was found (*κ* = 0.89). All data are summarized in Table 2.

**TABLE 2:** Performance evaluation in terms of different sample sources

Sample type	Gold standard	Sensitivity	Specificity	PPV	NPV
Saliva	Naso-oropharyngeal VTM	96.45% (95% CI: 92.82% to 98.56%)	92.44% (95% CI: 86.13% to 96.48%)	95.48% (95% CI: 91.84% to 97.54%)	94.02% (95% CI: 88.34% to 97.02%)
Naso-oropharyngeal VTM	Saliva	95.48% (95% CI: 91.59% to 97.91%)	94.02% (95% CI: 88.06% to 97.56%)	96.45% (95% CI: 92.97% to 98.24%)	92.44% (95% CI: 86.57% to 95.86%)

**FIG 3:**
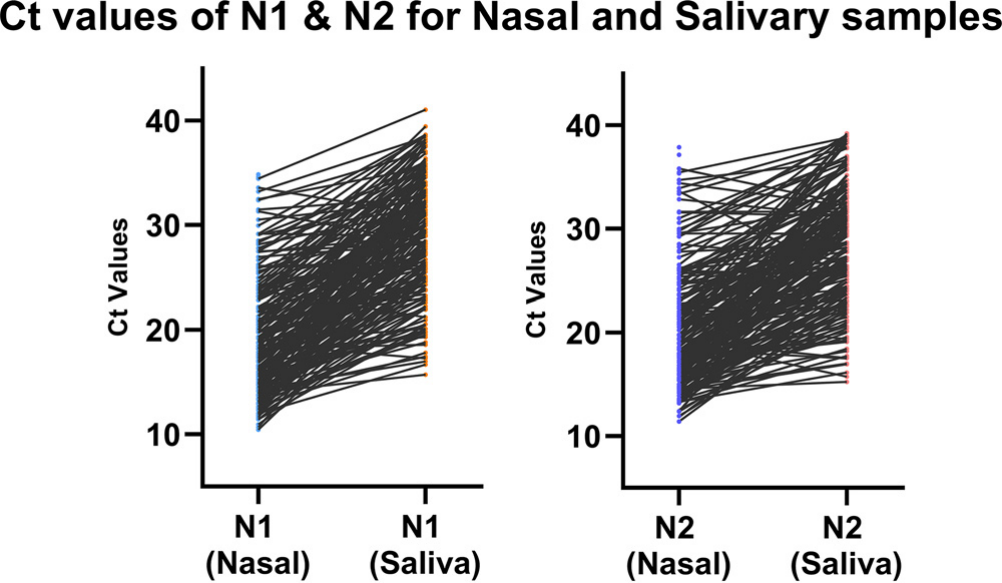
A line graph comparing the *C_T_* values for each target (N1 and N2) between nasal VTM and salivary sources.

The *C_T_* values of N1 and N2 were slightly higher in RT-PCR of salivary samples than in corresponding nasal VTM samples. These data have been depicted in Fig. 3, with a line graph indicating *C_T_* values of each sample by the terminal of each line.

The difference of *C_T_* values has also been portrayed through a Bland-Altman plot for both N1 and N2 targets in Fig. S1 and S2 in the supplemental material. The analysis showed a significant mean difference of *C_T_* values for N1 and N2 targets of about 10.0 and 8.5, respectively.

### Performance of SD Biosensor Standard Q COVID-19 antigen (Ag) test with VTM samples.

**FIG 4:**
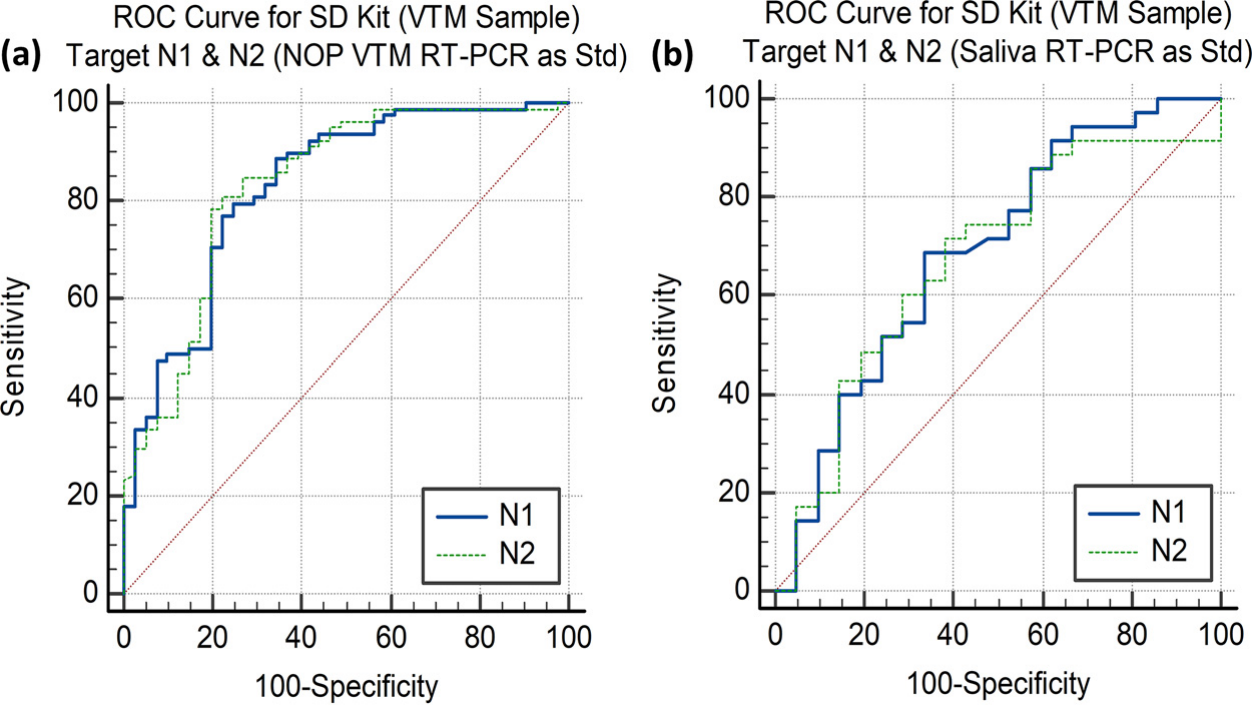
ROC curve for the SD Biosensor kit presenting the cut points where highest sensitivity and specificity can be attained for N1 and N2 targets with NOP VTM RT-PCR as standard (a) and saliva RT-PCR as standard (b); Std, standard.

Among the 207 patients from the first wave of COVID-19 infection, 119 tested positive by RT-PCR (57.49%) of NOP swabs in VTM. Only 78 patients tested positive by SD Biosensor Standard Q COVID-19 Ag test among the 119 RT-PCR-positive patients; and among 88 negative patients, 84 were negative by the rapid test. The kit’s sensitivity and specificity values were 65.55% (95% CI of 56.28% to 74.02%) and 95.46% (95% CI of 88.77% to 98.75%), respectively, as presented in Table 3. The PPV and NPV were 95.12% (95% CI of 88.12% to 98.09%) and 67.20% (95% CI of 61.43% to 72.49%), respectively. Performance (*κ* = 0.58) is summarized in the receiver operating characteristic (ROC) curve in Fig. 4a.

**TABLE 3:** The performance of the rapid test kits used in the two waves of COVID-19

Session	Device	Sample source	Gold standard	Sensitivity	Specificity	PPV	NPV
First wave	SD Biosensor Standard Q	NOP VTM	RT-PCR of NOP VTM	65.55% (95% CI: 56.28% to 74.02%)	95.46% (95% CI: 88.77% to 98.75%)	95.12% (95% CI: 88.12% to 98.09%)	67.20% (95% CI: 61.43% to 72.49%)
		NOP VTM	RT-PCR of saliva	62.50% (95% CI: 48.55% to 75.08%)	94.74% (95% CI: 85.38% to 98.90%)	92.11% (95% CI: 79.19% to 97.28%)	72% (95% CI: 64.59% to 78.38%)
Second wave	SD Biosensor Standard Q	Nasal swab	RT-PCR of NOP VTM	86.58% (95% CI: 80.03% to 91.61%)	98.61% (95% CI: 92.50% to 99.97%)	99.23% (95% CI: 94.85% to 99.89%)	78.02% (95% CI: 70.23% to 84.23%)
		Nasal swab	RT-PCR of saliva	83.92% (95% CI: 76.85% to 89.52%)	95.00% (95% CI: 86.08% to 98.96%)	97.56% (95% CI: 92.98% to 99.18%)	71.25% (95% CI: 62.92% to 78.35%)
	CareStart	NP swab	RT-PCR of NOP VTM	85.91% (95% CI: 79.27% to 91.06%)	98.61% (95% CI: 92.50% to 99.97%)	99.23% (95% CI: 94.81% to 99.89%)	77.17% (95% CI: 69.44% to 83.42%)
		NP swab	RT-PCR of saliva	83.22% (95% CI: 76.07% to 88.94%)	95.00% (95% CI: 86.08% to 98.96%)	97.54% (95% CI: 92.92% to 99.17%)	70.37% (95% CI: 62.14% to 77.46%)

Considering RT-PCR of saliva as the gold standard, 35 samples tested positive by SD Biosensor Standard Q COVID-19 Ag test out of 56 positive samples in the first session. The sensitivity and specificity values were slightly decreased to 62.50% (95% CI of 48.55% to 75.08%) and 94.74% (95% CI of 85.38% to 98.90%), respectively. The PPV and NPV were 92.11% (95% CI of 79.19% to 97.28%) and 72% (95% CI of 64.59% to 78.38%), respectively. The sensitivities at different *C_T_* values are summarized in Table 4 and Fig. 4b.

**TABLE 4:** Percent sensitivity of the kits at different *C_T_* values with respect to VTM and saliva RT-PCR as standard

Kit type	SD VTM	SD nasal	CareStart NP	SD VTM	SD nasal	CareStart NP
Standard	VTM	VTM	VTM	Saliva	Saliva	Saliva
*C_T_* < 18	94.7	97.8	97.8	–^*a*^	100.0	100.0
*C_T_* < 22	83.3	93.1	100.0	–^*a*^	100.0	100.0
*C_T_* < 26	66.7	73.3	66.7	75.0	89.7	89.7
All *C_T_* values	65.6	86.6	85.9	62.5	83.9	83.2

a –, no data.

### Performance of SD Biosensor Standard Q COVID-19 Ag test with direct nasal samples.

**FIG 5:**
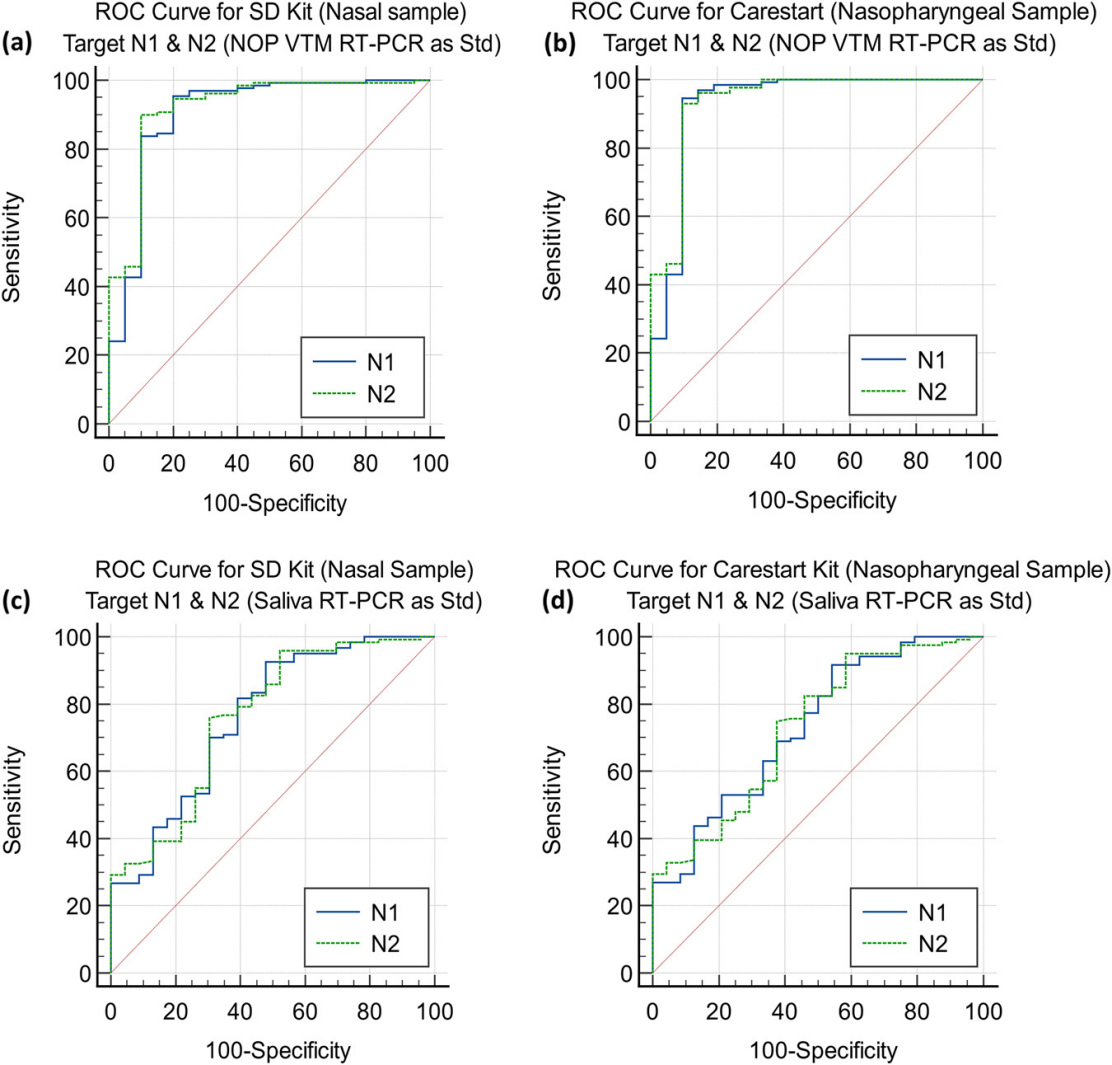
(a and b) ROC curve for SD Biosensor test kits using direct nasal samples (a) and ROC curve for CareStart test kits using direct NP samples (b) with NOP VTM RT-PCR as standard. (c and d) ROC curve for SD Biosensor test kits using direct nasal samples (c) and ROC curve for CareStart test kits using direct NP samples (d) with saliva RT-PCR as standard.

The SD Biosensor Standard Q COVID-19 Ag test kit was evaluated using nasal swabs from the second wave of COVID-19 infections. Of 221 symptomatic patients, 149 tested positive by RT-PCR, of which, 129 were positive by SD Biosensor Standard Q COVID-19 Ag test. The sensitivity and specificity values for the SD test were 86.58% (95% CI of 80.03% to 91.61%) and 98.61% (95% CI of 92.50% to 99.97%), respectively. The PPV was 99.23% (95% CI of 94.85% to 99.89%), and the NPV was 78.02% (95% CI of 70.23% to 84.23%). The performance (*κ* = 0.80) for the SD Biosensor Standard Q COVID-19 Ag test is described in Fig. 5a and Table 3.

The sensitivity and specificity values of this kit were 83.92% (95% CI of 76.85% to 89.52%) and 95.00% (95% CI of 86.08% to 98.96%), respectively, when the RT-PCR results of the saliva were considered the gold standard. The PPV and NPV were 97.56% (95% CI of 92.98% to 99.18%) and 71.25% (95% CI of 62.92% to 78.35%), respectively. The sensitivity levels are compared in Table 4 and Fig. 5c.

### Performance of the CareStart Ag test with direct nasopharyngeal samples.

The CareStart Ag rapid test kit was evaluated in terms of NP sample source from the same 221 symptomatic patients mentioned above, and out of those 149 RT-PCR-positive patients, 128 were found positive by CareStart Ag rapid test. The sensitivity and specificity values were 85.91% (95% CI to 79.27% to 91.06%) and 98.61% (95% CI of 92.50% to 99.97%), respectively. The PPV and NPV were 99.23% (95% CI of 94.81% to 99.89%) and 77.17% (95% CI of 69.44% to 83.42%), respectively. The performance (*κ* = 0.79) is described in Fig. 5b and Table 3.

Similarly, the sensitivity and specificity values of the kits were 83.22% (95% CI of 76.07% to 88.94%) and 95.00% (95% CI of 86.08% to 98.96%) compared to RT-PCR of saliva as the gold standard. Here, the PPV and NPV were 97.54% (95% CI of 92.92% to 99.17%) and 70.37% (95% CI of 62.14% to 77.46%). A detailed summary and comparison of sensitivities at different *C_T_* values are presented in Table 4 and Fig. 5d.

**FIG 6:**
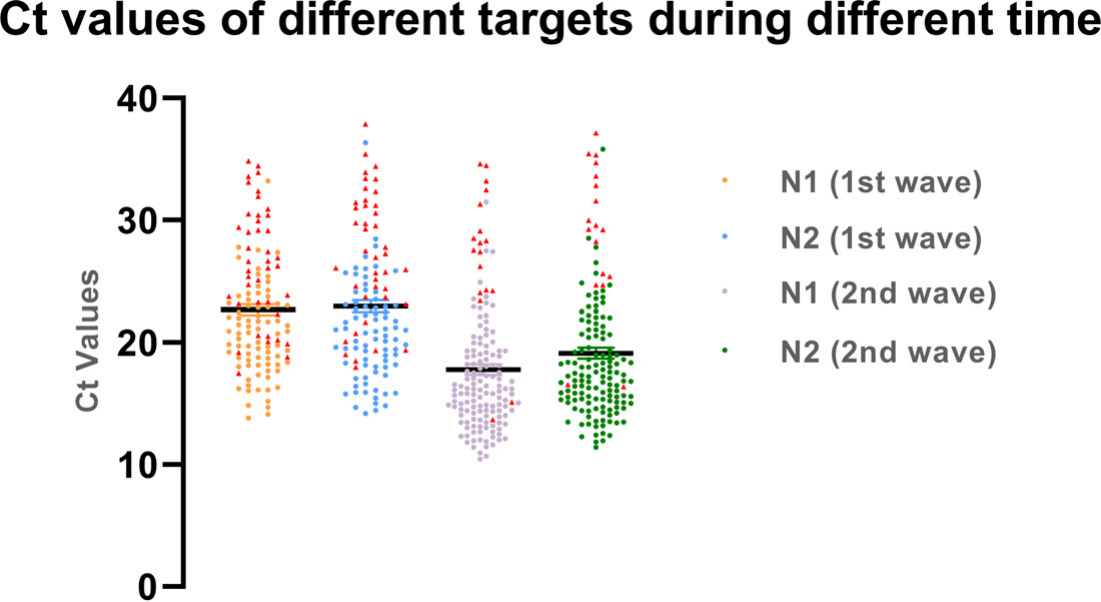
Scatter diagram showing the distribution of the *C_T_* values of the infected patients during the first and second waves of COVID-19. The RT-PCR-positive samples that were missed by RDT are shown by the red triangles. In the first wave, only the SD Biosensor kit was used, and both SD Biosensor and CareStart kits were used during the second wave.

The RDTs could not detect 6 samples with *C_T_* values less than 26 for the N1 and N2 targets in the second session. During the first wave, this number was 18. The RDTs mostly missed samples that had *C_T_* values greater than 26. Fig. 6 shows a scatter diagram, with the red triangles representing positive samples that the RDT missed. The true-positive and false-positive rates are summarized in the ROC curve in Fig. 2 and 3.

### Detection based on symptoms.

**FIG 7:**
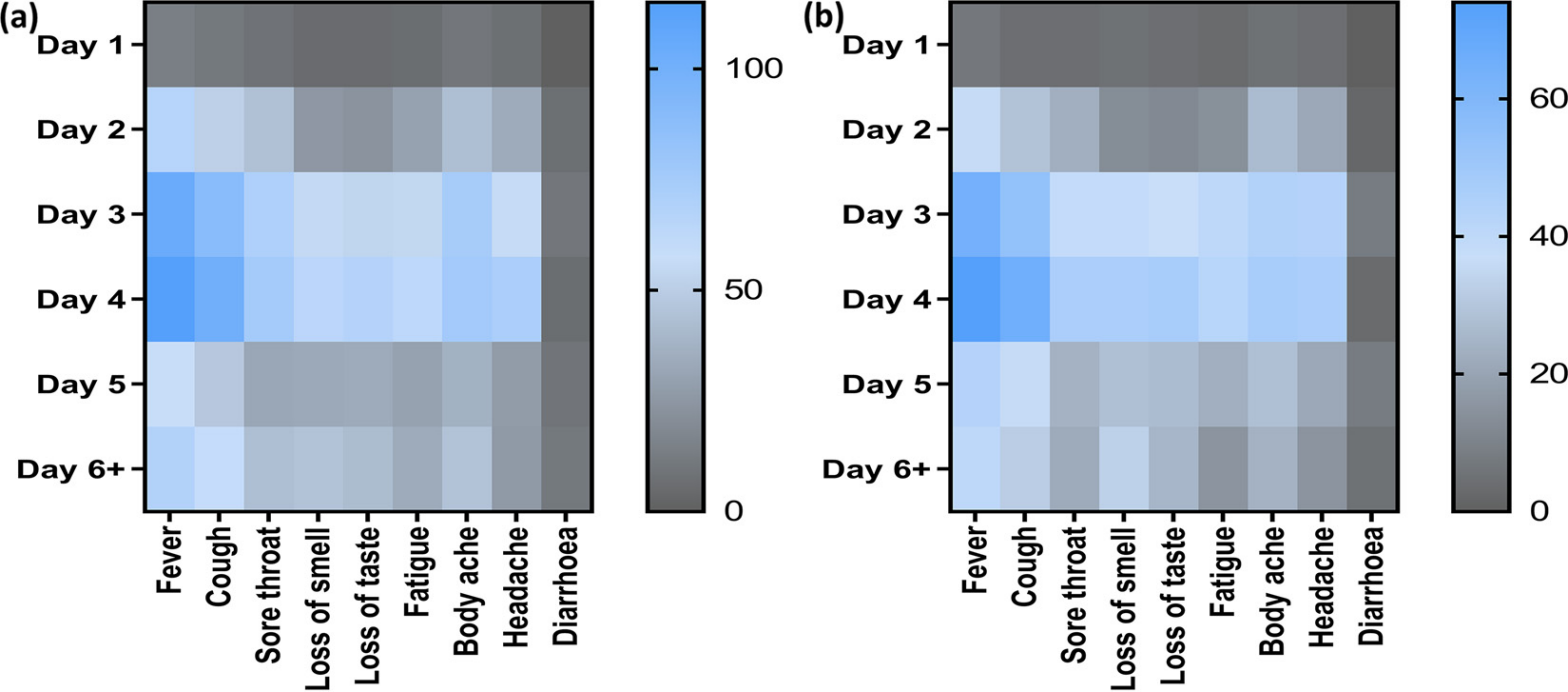
Heat map showing 9 major symptoms observed among the symptomatic patients. Each heat map contains day 1 to day 6 and later after onset of symptoms, with the right-sided bar representing the number of patients. (a and b) The total number of patients coming for testing (a) and the patients who were positive by rapid test only (b) are indicated.

Nine major symptoms, including fever, sore throat, loss of taste, cough, loss of smell, body aches, headache, fatigue, and diarrhea, were observed and are summarized through a heat map in Fig. 7. The frequency of patients is depicted with a bar in each heat map, and the onset of symptoms was distributed between day 1 and day 6 and later.

### Variant-based detection.

**FIG 8:**
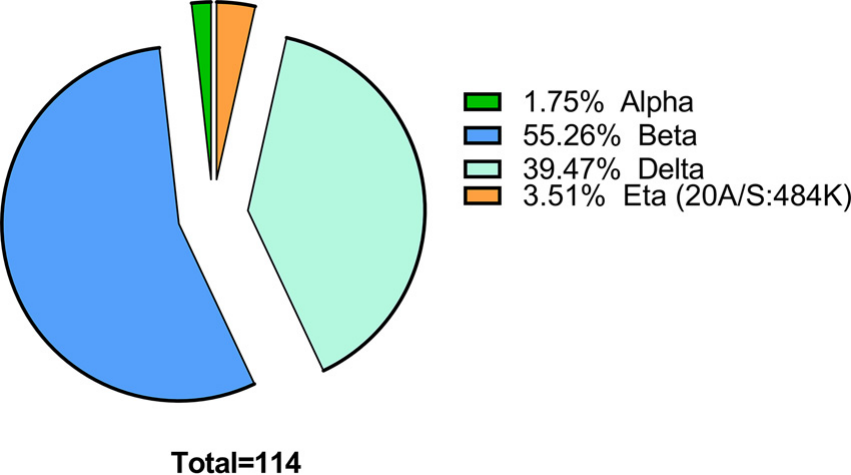
Pie chart of the circulating variants during the two COVID-19 waves.

Among the 114 samples sent for Sanger sequencing, 2 were the Alpha (United Kingdom) variant (1.75%), 63 were the Beta (South African) variant (55.26%), 45 were the Delta (Indian) variant (39.47%), and 4 were the Eta (20A/S:484K probably Nigerian) variant (3.51%). This indicates that RDT had the capacity to detect major circulating variants of SARS-CoV-2 (Fig. 8).

## DISCUSSION

Several prospective and retrospective studies have been conducted to evaluate the practicality of the rapid diagnostic test devices in various platforms worldwide (13–15). Some studies evaluated only a single testing device in limited sample size (16, 17), whereby a few studies have investigated multiple rapid test devices simultaneously (18, 19). In most cases, the sample size is very small for prospective studies (20) and large for retrospective studies (21). Regarding specimen source (nasal and/or saliva) evaluation, only a small handful of studies exist, and this substantiates further studies (22, 23). In this study, we tried to combine all the knowledge gaps in terms of rapid tests and sample sources, rendering some important outcomes for further implementation. For the rapid test analysis, only 6 RT-PCR-positive samples with *C_T_* values less than 27 were missed by the rapid tests during the second wave of COVID-19, where direct nasal or NP swab samples were collected first for rapid testing followed by a second NP sample collection in VTM for RT-PCR testing. These 6 false-negative samples could be explained by improper collection by the health care professional or the dried nostrils of the patients often reported by the health care staff. Interestingly, many samples with *C_T_* values less than 27 were missed in the first session, where the NP VTM was sent to a central lab within 6 h for testing with rapid devices. This indicates the poor performance of the rapid tests when the specimen source is nasal VTM rather than direct nasal or NP swabs. The initial plan to collect only NP VTM samples during the first wave was to reduce the number of swab collections for better compliance among patients. The poor performance eventually led us to the multiple swab collection method for individual kit testing simultaneously in the second wave following the manufacturer’s instructions for use.

One recent study evaluated the performance of the SD Biosensor kit and compared it with the PanBio kit. The study found that the PanBio and SD Biosensor rapid Ag test devices have sensitivities of 87.7% and 90.4%, respectively, when tests were performed within 7 days after the onset of symptoms, and sensitivity falls notably after 7 days (24). In another retrospective study, the SD Biosensor kit was used on 65 stored positive samples and was compared with the Allplex (Seegene) kit (25). The sensitivity was found to be 89.2%, although most of the samples there had a *C_T_* value less than 30. Another study recruited 1,465 patients for Roche/SD Biosensor rapid tests, among which only 141 patients were RT-PCR positive. The overall sensitivity was found to be only 65.3% even in this small sample size (26). In this study, the authors showed that the sensitivity can be raised up to 84.4% if the cutoff for *C_T_* value was reduced to 30 cycles only. A systematic review by Cochrane databases showed that most of the COVID-19 Ag-based rapid diagnostic test studies suffer from methodological concerns, and there is a considerable risk for sample bias (27). Considering this, our study has robust information composed of two different COVID-19 waves along with the analysis of multiple specimen sources in several rapid tests. The overall sensitivity found in our study for both SD Biosensor and CareStart RDTs (i.e., 86.24%) was high enough to be used in a clinical setting specially for recent infection. The performance also substantiates the importance of RDTs for early and rapid diagnosis in clinical settings.

In terms of salivary samples, a large study with symptomatic and asymptomatic 23,740 salivary sample donors, yielding 465 positive cases, concluded that the salivary sample is an undervalued resource, and the accuracy was greater than 99% and the sensitivity was as low as 1 to 10 viral copies/μL (22). Another study evaluated saliva and NP samples, and the sensitivity was found to be 85.2% for saliva when NP was the standard and was 94.5% for NP with saliva as the standard. The study also found that saliva is a poor choice after a long duration of symptoms but can be a good alternative in children (28). The study was conducted in a children-based facility, and the poor performance can often be explained by improper collection. Often, saliva has been recommended as superior over NP swabs (29). In comparison, our study has a sensitivity of 96.45% and 95.48% for salivary and NP sample with standard as vice versa using stored samples in −80°C. Interestingly, one of the samples, positive by both RDT and negative with RT-PCR using NP VTM, was found to be positive by salivary RT-PCR. This high sensitivity even in these archived samples indicates that salivary samples can be an excellent alternative to NP, with an advantage of being noninvasive, which is beneficial in unassisted collection from pediatric, geriatric, and noncompliant patients.

One of the major limitations of this study is that we could not test both SD Biosensor and CareStart rapid devices for all the samples during the first wave of COVID-19 due to a shortage of kit supplies worldwide; therefore, we could not compare SD Biosensor with CareStart performance for samples collected in NP VTM. Additionally, we also tried to test salivary samples with rapid kits, but in most cases, the buffer did not pass through the nitrocellulose strips of the test cassettes. Therefore, we could not evaluate the performance of salivary samples using rapid tests. The major flaw of this study design is not being able to test the salivary samples using RT-PCR as soon as the sample is collected. Most of these samples have been stored for months before performing the RT-PCR tests, and hence the high *C_T_* values in Fig. 3 and Fig. S1 and S2 in the supplemental material can be explained due to multiple freeze-thaw cycles.

### Conclusion.

Our study tried to cover various rapid testing kit platforms and specimen types. Overall sensitivity obtained in this study suggests that rapid testing using direct nasal or direct NP swabs is a suitable option specially for acute infection diagnosis in a clinical setting. The tremendous burden of COVID-19 RT-PCR can be reduced with these inexpensive, rapid, and easy-to-use rapid testing devices. It is also evident from our study that the RT-PCR tests that mostly use naso-oropharyngeal (NOP) VTM samples can also be well substituted with RT-PCR of saliva, which is very easy to collect.

## MATERIALS AND METHODS

### Ethical considerations.

Informed consent was obtained from each enrolled adult patient or parent or legal guardian for children aged 2 to 10 years old and children between 11 and 17 years old. In addition, verbal assent was obtained from children between 11 and 17 years old. The study was conducted with prior approval from the Institutional Ethical Review Committee (ERC) under the institutional review board (IRB) of the International Center for Diarrheal Disease Research, Bangladesh (icddr,b), with study protocol number PR-20133.

### Patients.

Symptomatic patients coming to the diagnostic facility of the icddr,b for a COVID-19 RT-PCR test and residing in Dhaka, Bangladesh, were enrolled in the study. The study was conducted in two sessions comprising two COVID-19 waves from 3 February to 1 June and from 13 April to 8 August 2021. Approximately 207 and 221 patients with fever and any other COVID-19 symptoms, including sore throat, loss of taste, cough, loss of smell, body aches, headache, fatigue, and diarrhea (verified by a physician), were enrolled in two sessions, respectively. Proper consent was obtained from individual patients and assent where necessary.

### Sample collection.

In the first session of the study, naso-oropharyngeal swab samples were collected from the enrolled participants in 3 mL of VTM by expert health care professionals, and samples were later assessed on a rapid diagnostic test (RDT) with the SD Biosensor Standard Q COVID-19 Ag kit within 2 h and RT-PCR within 24 h of sample collection. During the second session, two different rapid diagnostic tests were performed on the spot from nasal and nasopharyngeal (NP) swabs using an SD Biosensor Standard Q COVID-19 Ag kit and CareStartCOVID-19 Ag test, respectively, for each patient. NOP swabs were also collected from corresponding participants to perform the gold standard RT-PCR. Salivary samples were collected in both sessions from symptomatic patients donating willingly. The methodology followed in this study is relatively large and is depicted in Fig. 1 as a flowchart.

### Rapid diagnostic tests.

The SD Biosensor Standard Q COVID-19 Ag and CareStart COVID-19 Ag kits are rapid immunochromatographic tests that qualitatively detect SARS-CoV-2 nucleocapsid protein antigen in human nasal and anterior nasopharyngeal swab samples, respectively. Both test kits work on the same principle (9). In brief, the sample is eluted into the extraction tube (from VTM or direct nasal and NP samples), and extracted swab samples are then added to the sample well of the test device kit that contains a control and a test line. The sample then migrates through the test strip, and the SARS-CoV-2 viral antigen, if present, binds to the anti-SARS-CoV-2 nucleocapsid protein antibodies conjugated to indicator, forming an immunocomplex. The positive sample contains a colored test line, with the presence of a colored control line as an indicator of test validity.

### RT-PCR for COVID-19 diagnosis.

RT-PCR of NOP swabs from prospective patients collected in VTM was performed in an ISO 15189- and ISO 15190-approved facility here at icddr,b. N1 and N2 gene targets with ribonuclease P (RNaseP) as human internal control were amplified using primer probes recommended by the Centers for Disease Control and Prevention (30). The MagMAX viral/pathogen II (MVP II) nucleic acid isolation kit was used for NP sample extraction with an automated high-throughput extraction system. In brief, TaqPath 1-step RT-qPCR master mix was used in a 20-μL reaction mix containing 5 μL of template. The ABI 7500 Fast DX thermocycler was used for RT-PCR. The reaction includes initial cDNA synthesis at 55°C for 10 min, denaturation at 95°C for 1 min, and subsequent 45 cycles of denaturation at 95°C for 10 s and annealing and/or elongation at 55°C for 30 s (31).

For salivary samples, a Qiagen viral RNA kit was used for extraction purposes, and an UltraPlex 1-step ToughMix was used as master mix during RT-PCR. 5 μL of template was used similarly in a 20-μL reaction mix targeting the same genes. RT-PCR was performed with a slightly modified reaction condition that includes cDNA synthesis at 50°C for 10 min and an initial denaturation at 95°C for 1 min, with subsequent 45 cycles of denaturation at 95°C for 10 s and annealing and/or elongation at 55°C for 30 s on a Bio-Rad CFX Opus 96 thermocycler.

### Sanger sequencing and clade identification.

Sanger sequencing was performed to evaluate the capacity of the rapid tests to detect various circulating variants. The spike protein gene was sequenced using ARTIC nCoV-2019 V3 panel primer sets for SARS-CoV-2 variants of concern (VOCs). An ABI 3500 XL genetic analyzer was used for Sanger sequencing of 114 samples with *C_T_* values less than 27 among 268 RT-PCR-positive samples (32). To examine the raw sequence data, BLAST searches were used, and to map contigs, the SeqMan program was used with default settings. Nextclade v1.5.2 was used to discover the SARS-CoV-2 clade (accessed on 18 March 2022) (33).

### Data analysis.

GraphPad Prism 8.0.2 and Stata version 15.1 were used for the statistical analysis and graph representation. MedCalc statistical software version 20.013 was used to calculate sensitivity, specificity, positive predictive values (PPVs), negative predictive value (NPVs), and respective confidence intervals along with preparation of graphs and plots (Bland-Altman plot, receiver operating curves [ROC], etc.).

### Data availability.

SARS-CoV-2 nucleotide sequences have been deposited in GenBank (accession numbers OP964845 to OP964914). All other data supporting the findings of this article are available from the corresponding author upon reasonable request.
