# Erratum for Sazed et al., “Direct Nasal Swab for Rapid Test and Saliva as an Alternative Biological Sample for RT-PCR in COVID-19 Diagnosis”

**DOI:** 10.1128/spectrum.04014-23

**Published:** 2024-01-22

**Authors:** Saiful Arefeen Sazed, Mohammad Golam Kibria, Md Fahad Zamil, Mohammad Sharif Hossain, Jeba Zaman Khan, Rifat Tasnim Juthi, Mohammad Enayet Hossain, Dilruba Ahmed, Zannatun Noor, Rashidul Haque, Mohammad Shafiul Alam

## ERRATUM

Volume 10, no. 6, e01998-22, 2022, https://doi.org/10.1128/spectrum.01998-22. The last sentence of the abstract should read “Besides, the analysis also signifies that RT-PCR of saliva can be a used as a replacement for RT-PCR of NOP VTM, providing ease of sample collection with a non-invasive method.”

