# Supplementary material for: Direct Nasal Swab for Rapid Test and Saliva as an Alternative Biological Sample for RT-PCR in COVID-19 Diagnosis
Source: Microbiol Spectr. 2022 Dec 1;10(6):e01998-22. doi: 10.1128/spectrum.01998-22 (PMC9769842; doi:10.1128/spectrum.01998-22)
Supplement: Supplemental file 1 — Supplemental material. Download spectrum.01998-22-s0001.pdf, PDF file, 0.2 MB [file spectrum.01998-22-s0001.pdf]

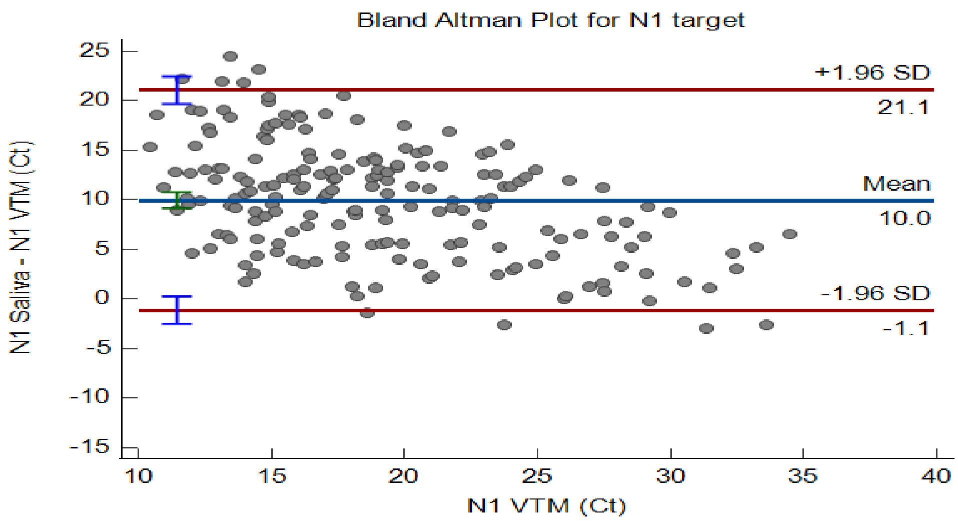

S1: Bland Altman plot comparing the difference of Ct values between nasal VTM and salivary sample source for N1 target. The X axis contains the Ct values for nasal VTM samples and the Y axis presents the difference between the Ct values of salivary and nasal VTM.

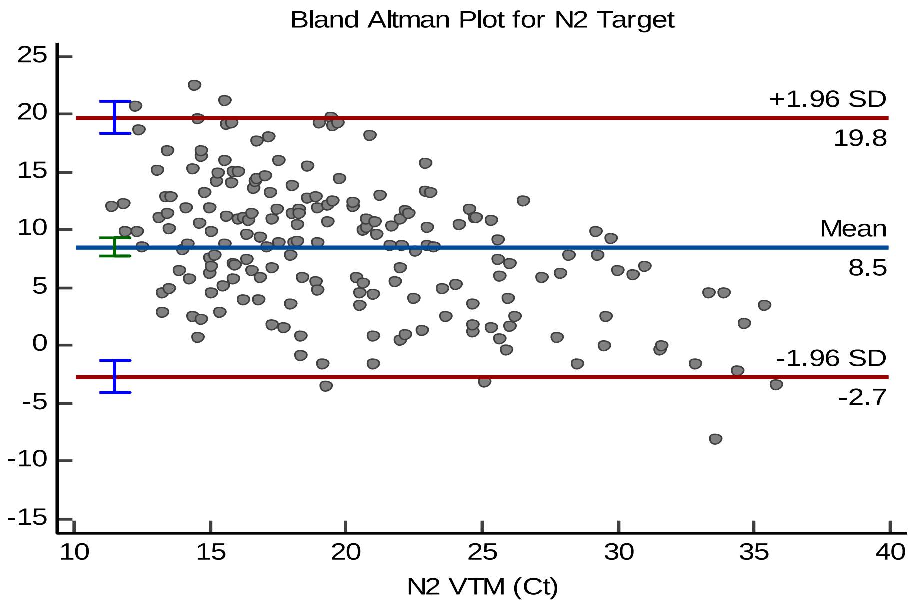

S2: Bland Altman plot comparing the difference of Ct values between nasal VTM and salivary sample source for N2 target. The X axis contains the Ct values for nasal VTM samples and the Y axis presents the difference between the Ct values of salivary and nasal VTM.
